# The calmodulin-like proteins AtCML4 and AtCML5 are single-pass membrane proteins targeted to the endomembrane system by an N-terminal signal anchor sequence

**DOI:** 10.1093/jxb/erw101

**Published:** 2016-03-29

**Authors:** Henning Ruge, Sandra Flosdorff, Ingo Ebersberger, Fatima Chigri, Ute C. Vothknecht

**Affiliations:** ^1^Department of Biology I, Faculty of Biology, LMU Munich, Großhaderner Straße 2–4, D-82152 Planegg, Germany; ^2^Department for Applied Bioinformatics, Institute for Cell Biology and Neuroscience, Goethe-University Frankfurt, Max-von-Laue-Straße 13, 60438 Frankfurt am Main, Germany; ^3^Center for Integrated Protein Science (Munich) at the Department of Biology I, Faculty of Biology, LMU Munich, D-81377 Munich, Germany

**Keywords:** Arabidopsis, calcium signalling, calmodulin, endomembrane system, endosomes, Golgi, vesicle transport.

## Abstract

Calmodulins (CaMs) are important mediators of Ca^2+^ signals that are found ubiquitously in all eukaryotic organisms. Plants contain a unique family of calmodulin-like proteins (CMLs) that exhibit greater sequence variance compared to canonical CaMs. The *Arabidopsis thaliana* proteins AtCML4 and AtCML5 are members of CML subfamily VII and possess a CaM domain comprising the characteristic double pair of EF-hands, but they are distinguished from other members of this subfamily and from canonical CaMs by an N-terminal extension of their amino acid sequence. Transient expression of yellow fluorescent protein-tagged AtCML4 and AtCML5 under a 35S-promoter in *Nicotiana benthamiana* leaf cells revealed a spherical fluorescence pattern. This pattern was confirmed by transient expression in Arabidopsis protoplasts under the native promoter. Co-localization analyses with various endomembrane marker proteins suggest that AtCML4 and AtCML5 are localized to vesicular structures in the interphase between Golgi and the endosomal system. Further studies revealed AtCML5 to be a single-pass membrane protein that is targeted into the endomembrane system by an N-terminal signal anchor sequence. Self-assembly green fluorescent protein and protease protection assays support a topology with the CaM domain exposed to the cytosolic surface and not the lumen of the vesicles, indicating that AtCML5 could sense Ca^2+^ signals in the cytosol. Phylogenetic analysis suggests that AtCML4 and AtCML5 are closely related paralogues originating from a duplication event within the Brassicaceae family. CML4/5-like proteins seem to be universally present in eudicots but are absent in some monocots. Together these results show that CML4/5-like proteins represent a flowering plant-specific subfamily of CMLs with a potential function in vesicle transport within the plant endomembrane system.

## Highlight

AtCML4 and AtCML5 are single-pass membrane proteins localized to the interphase between Golgi and the endosomal system. They might provide a basis for calcium regulation of endosomal vesicle transport.

## Introduction

Plants have evolved to adapt to their specific habitats, resulting in optimized growth and development. However, even within the borders of their habitats, plants are regularly exposed to rapid and often unpredictable abiotic and biotic stresses. Because they cannot escape these conditions, they contain a toolbox of sensors, sensor transducers, and target proteins that allows them to react to certain stimuli on a cellular level (reviewed in Kudla *et al.*, 2010). Within this context, Ca^2+^ is a secondary messenger that plays an important role in mediating environmental cues into an appropriate cellular response. Many different biotic and abiotic stimuli are known to cause temporal and spatial changes in intracellular Ca^2+^ concentration that are recognized by specific Ca^2+^ sensors (DeFalco *et al.*, 2010; Dodd *et al.*, 2010; Kudla *et al.*, 2010). A ubiquitous sensor of Ca^2+^ in eukaryotic cells is calmodulin (CaM), a highly conserved protein that, in its canonical form, contains four Ca^2+^-binding EF-hand domains and is otherwise devoid of additional functional domains (McCormack and Braam, 2003; Yang and Poovaiah, 2003). Calcium binding induces a conformational change in the CaM molecule that allows it to bind to a large and diverse group of target proteins (Lewit-Bentley and Rety, 2000). CaM has been shown to affect many different cellular processes, including changes in gene expression, activation of ion channels, initiation of phosphorylation cascades, and the direct alteration of metabolic enzymes (reviewed in White and Broadley, 2003; Yang and Poovaiah, 2003; Bouche *et al.*, 2005).

Plants, but not animals or fungi, contain a large family of so-called CaM-like proteins (CMLs) that differ from canonical CaMs in that they have a higher variability in length, sequence, and number of EF-hand domains (reviewed in Bender and Snedden, 2013). Over 50 CMLs are encoded in the genome of *Arabidopsis thaliana* (McCormack *et al.*, 2005) and orthologues of these are found in other plants. They fall into various subfamilies that appear to be differentially distributed throughout the green lineage (Boonburapong and Buaboocha, 2007; Zhu *et al.*, 2015). The CML family is already remarkably expanded in the algae, ferns, and gymnosperms, but is especially prominent in the angiosperm lineage (Bender and Snedden, 2013).

As sessile organisms, land plants cannot escape changes in their environment but have to find an adequate response on the cellular level. The emergence of the vast number of CMLs in land plants has thus been associated with their need to process various environmental cues. It was shown that several CMLs display alterations of gene expression in response to different abiotic and biotic stimuli or hormones (recently reviewed in Zhu *et al.*, 2015). Also, several CMLs display elongated N- or C-termini that might represent targeting sequences. Along this line, experimental evidence has been obtained for the localization of CaMs or CMLs to cellular sub-compartments such as the vacuole, chloroplasts, mitochondria, and peroxisomes (Yamaguchi *et al.*, 2005; Chigri *et al.*, 2006; Chigri *et al.*, 2012; Bender and Snedden, 2013). Evidence for Ca^2+^/CaM regulation of processes in various sub-compartments has also been reported (Jarrett *et al.*, 1982; Sauer and Robinson, 1985; Miernyk *et al.*, 1987; Pou de Crescenzo *et al.*, 2001; Yang and Poovaiah, 2002; Chigri *et al.*, 2005; Bussemer *et al.*, 2009; Kuhn *et al.*, 2009). In peroxisomes, CaM has been associated with catalase activity as well as with the regulation of DEG15 protease activity (Yang and Poovaiah, 2002; Dolze *et al.*, 2013). In mitochondria and chloroplasts, CaM regulation has been associated with NAD(H) kinase activity (Anderson *et al.*, 1980; Turner *et al.*, 2004) as well as several AAA^+^-ATPases of unknown function (Reddy *et al.*, 2002; Bussemer *et al.*, 2009). It was also shown that translocation of nuclear-encoded proteins into both organelles is affected by Ca^2+^/CaM (Chigri *et al.*, 2005; Kuhn *et al.*, 2009). In chloroplasts, this is mediated from the inside of the organelle by Tic32, a component of the inner membrane protein import translocon (Chigri *et al.*, 2006). Thus, the extent of CMLs might not only allow differential expression of these Ca^2+^ sensors in different tissues and at different developmental stages, but also facilitate targeting to various subcellular compartments. However, for most CMLs, the subcellular localization has not yet been established and their individual function within the calcium signalling network is not well understood.

In this work, the subcellular localization of two closely related CMLs from *A. thaliana*, AtCML4 and AtCML5, was analysed. They are targeted into the plant endomembrane system by an N-terminal signal anchor sequence and are localized in the interphase between Golgi and the endosomal system. Their C-terminal CaM domain is exposed to the cytosolic surface, indicating that they could sense Ca^2+^ signals in the cytosol. They possess typical characteristics of canonical CaMs that should enable them to sense changes in Ca^2+^ concentration and affect cellular processes in a Ca^2+^-dependent manner. AtCML4 and AtCML5 might thus provide a basis for Ca^2+^ regulation of endosomal vesicle transport.

## Material and methods

### Molecular cloning and construction of expression plasmids

35S-promotor driven transient expression of YFP fusion proteins was performed by *Agrobacterium* infiltration of tobacco leaf cells. To that end, the entire coding sequences of *AT2G43290* (*AtCML5*), *AT3G59440* (*AtCML4*), and *At1g66410* (*AtCAM4*) as well as variants thereof were cloned N-terminally to the YFP sequence into the plant expression vector pBIN19 (Datla *et al.*, 1992). For self-assembly GFP (saGFP) analysis (Cabantous *et al.*, 2005; Machettira *et al.*, 2011), the entire coding sequences of *AT2G43290* and *AT3G59440* as well as different control proteins were cloned into either pBIN19-saGFP_1–10_ or pBIN19-saGFP_11_ (a kind gift from Dr Stael, formerly of the University of Vienna), thereby creating N-terminal fusions to either the first 10 or the 11th beta-sheet of GFP. At1g66410 (AtCAM4) was used as a cytosolic marker (Cyt-saGFP_1–10_ + Cyt-saGFP_11_) and the chloroplast small outer envelope protein OEP7 (Mehlmer *et al.*, 2012) was used to mark the cytosolic surface of chloroplasts (OEP7-saGFP_1–10_). For transient expression of YFP fusion protein by PEG-mediated transformation of Arabidopsis protoplast under the endogenous promoter, 1754 base pairs of the 5′ UTR of AtCML4 and 1031 base pairs of the 5′ UTR of AtCML5 as well as their respective coding sequences were fused to the YFP coding sequence and cloned into pGREENII (Hellens *et al.*, 2000). An overview of all primers used as well as the final constructs is presented in Supplementary Tables S1 and S2.

### Transient expression in tobacco leaves and Arabidopsis protoplasts

*Agrobacterium*-mediated transformation of tobacco leaf cells was performed as described in Voinnet *et al.* (2003) with the *Agrobacterium* strain LBA1334. Protoplasts were isolated out of leaf tissue 48h after transformation as described in Koop *et al.* (1996) and further analysed using a Leica TCS SP5 confocal laser scanning microscope. The mCherry-fused marker proteins ER-mCherry (SP-AtWAK2-mCherry-HDEL), Golgi-mCherry (GmMAN1_1–49_-mCherry) and ARA6-mCherry were used for single and double transformation. All of these markers are expressed under the 35S-promoter and their correct localization under these conditions is well established (Ueda *et al.*, 2001; Nelson *et al.*, 2007). To detect mitochondria, protoplast suspensions were incubated for 30min at room temperature with 125nM of MitoTracker (RedCMX Ros Invitrogen, 1mM stock in dimethyl sulfoxide) in the appropriate protoplast incubation media. Image processing was performed using the Leica Application Suite for Advanced Fluorescence. For extraction experiments, tobacco leaf cells were also transformed with pBIN-OEP7-YA (Mehlmer *et al.*, 2012), a fusion protein with OEP7 that exposes the YFP-aequorin tag to the cytosol. Isolation and transformation of Arabidopsis mesophyll cell protoplasts was performed according to Yoo *et al.* (2007).

### Isolation of microsomal fractions and chloroplasts

Microsomal fractions and chloroplasts were isolated from tobacco leaves transiently expressing various YFP or mCherry fusion proteins. Expression of the proteins in leaf mesophyll cells was confirmed by fluorescence microscopy 48h after *Agrobacterium* infiltration. All following steps were performed at 4°C. Chloroplast isolation was performed according to Mehlmer *et al.* (2012). For the isolation of microsomal fractions, leaves were homogenized in 50mM Tris-HCl, pH 7.5, 1mM EDTA, and 0.5M sucrose and filtered through a 30 µm diameter nylon mesh. The microsomal fraction was further enriched by differential centrifugation for 10min at 4°C and 4200g (to pellet chloroplasts), followed by 10min at 4°C and 10 000g (to pellet mitochondria and nuclei), and finally for 1h at 4°C and 100 000g. The supernatant of the last centrifugation step was discarded and the pellet re-suspended in 10mM Tris-HCl, pH 7.5, 5mM EDTA, and 0.25M sucrose for membrane extraction assays and sucrose density gradient analysis, or in 10mM Tris-HCl, pH 7.5, 0.25M sucrose, and 2mM CaCl_2_ for thermolysin treatment.

### Membrane extraction, thermolysin treatment, and sucrose density gradient

For further separation, isolated microsomal fractions were placed on top of a 20–50% continuous sucrose density gradient and centrifuged for 16h at 4°C and 100 000g. Subsequently, 500 µl fractions were collected from the top to the bottom of the tube and analysed by SDS-PAGE and western blotting. For membrane extraction, isolated microsomal fractions and chloroplasts were centrifuged for 10min at 289 000g and 4200g, respectively. The pellets were re-suspended in 10mM Tris, pH 7.5, 5mM EDTA, and 0.25M sucrose containing either 0.5M NaCl, 0.1M Na_2_CO_3_, 6M urea, or 2% lithium dodecyl sulfate (LDS), and incubated for 20min at room temperature for the urea-containing samples or 4°C for all other samples. Afterwards, the reactions were centrifuged for 10min at 289 000g and 4200g, respectively, and pellets were re-suspended in SDS-PAGE sample buffer and analysed by SDS-PAGE and western blotting. For the protease protection assays, thermolysin was added to the microsomal fractions to a final concentration of 2 µg/µl. After 20min of incubation at 4°C, EDTA was added to a final concentration of 5mM to stop the reaction. After centrifugation for 10min at 289 000g, the pellets were resuspended in SDS-PAGE sample buffer and analysed by SDS-PAGE and western blotting. Western blot analyses was performed using antisera against RFP and GFP (ChromoTek GmbH, Germany), aequorin (Abcam, UK), as well as SMT1 and Arf1 (Agrisera AB, Sweden).

### Sequence alignment and phylogenetic tree construction

Accession numbers were obtained from the EMBL/GenBank data libraries and are listed in Supplementary Table S3. Sequences were aligned with MAFFT version 7 (http://mafft.cbrc.jp/alignment/server/index.html, accessed 4 March 2016) using the L-INS-i algorithm (Katoh *et al.*, 2002). Shading of the alignments was performed with BOXSHADE 3.31 (http://mobyle.pasteur.fr, accessed 4 March 2016). Maximum likelihood phylogeny trees were reconstructed with PhyML 3.1/3.0 aLRT (http://www.phylogeny.fr, accessed 4 March 2016) using the JTT substitution model (Jones *et al.*, 1992). We modelled substitution rate heterogeneity across sites with a gamma distribution allowing for a fraction of invariant sites. Branch support was assessed using a non-parametric bootstrap approach with 304 repetitions.

## Results

### AtCML4 and AtCML5 are localized in the endomembrane system

**Fig. 1.**
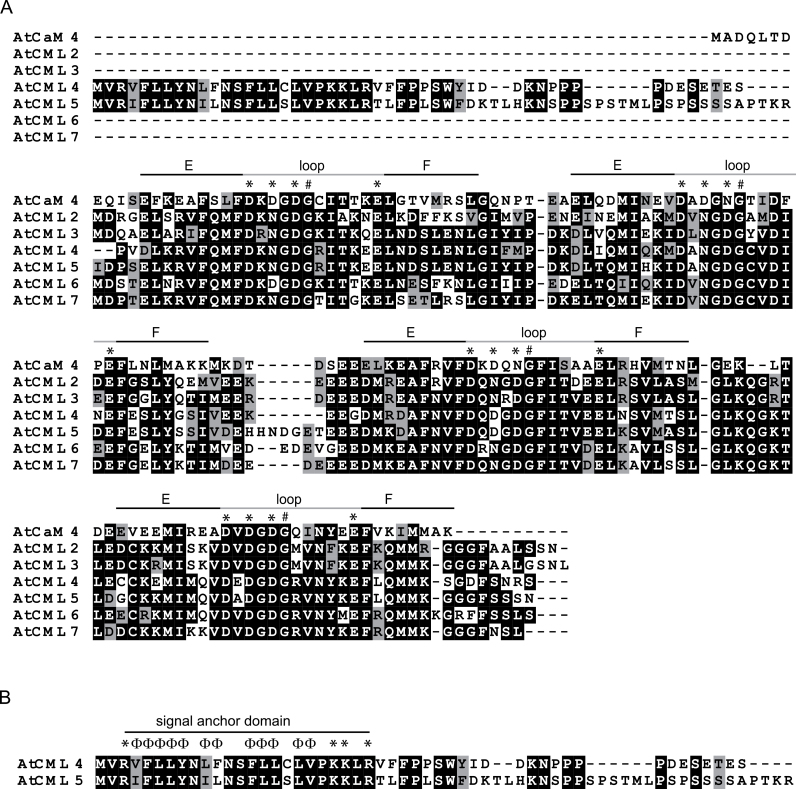
(**A**) Amino acid sequence alignment of AtCML2–7 and AtCaM4. Black boxes indicate identical amino acid residues while grey boxes indicate a conserved amino acid substitution. Black and grey bars above the sequence denote the four EF-hands with their characteristic 29-residue helix-loop-helix topology. Asterisks mark conserved residues involved in Ca^2+^ coordination, while the hash indicates a conserved glycine residue required for the conformational flexibility of the backbone. (**B**) Distribution of charged (*) and hydrophobic (Φ) residues within the signal anchor sequence of AtCML4 and AtCML5.

AtCML4 and AtCML5 are two closely related paralogues residing in CML subfamily VII. Similar to canonical CaMs they have a CaM domain comprising the characteristic double pair of EF-hands (Fig. 1A, marked). Both proteins possess an N-terminal extension that distinguishes them from other members of this subfamily and from the canonical CaMs (Fig. 1A). Taking AtCaM4 as a reference, these extensions span 40 amino acids in AtCML4 and 53 amino acids in AtCML5. A subsequent comparison of the two N-terminal domains revealed a marked sequence similarity, especially in the first 28 amino acids, hinting towards a shared evolutionary descent and/or a similar function. Initial support of the latter hypothesis comes from an *in silico* screen for signal peptides. Different tools unanimously predict a subcellular targeting sequence at the N-terminus of both proteins. However, there was no unanimous agreement with respect to the predicted intracellular location of the proteins (Supplementary Table S4). According to the Aramemnon Plant Membrane Protein Database (http://aramemnon.botanik.uni-koeln.de, accessed 3 March 2016), targeting of AtCML5 to the chloroplast or the secretory pathway is equally likely. In the case of AtCML4 the secretory pathway is clearly favoured.

**Fig. 2.**
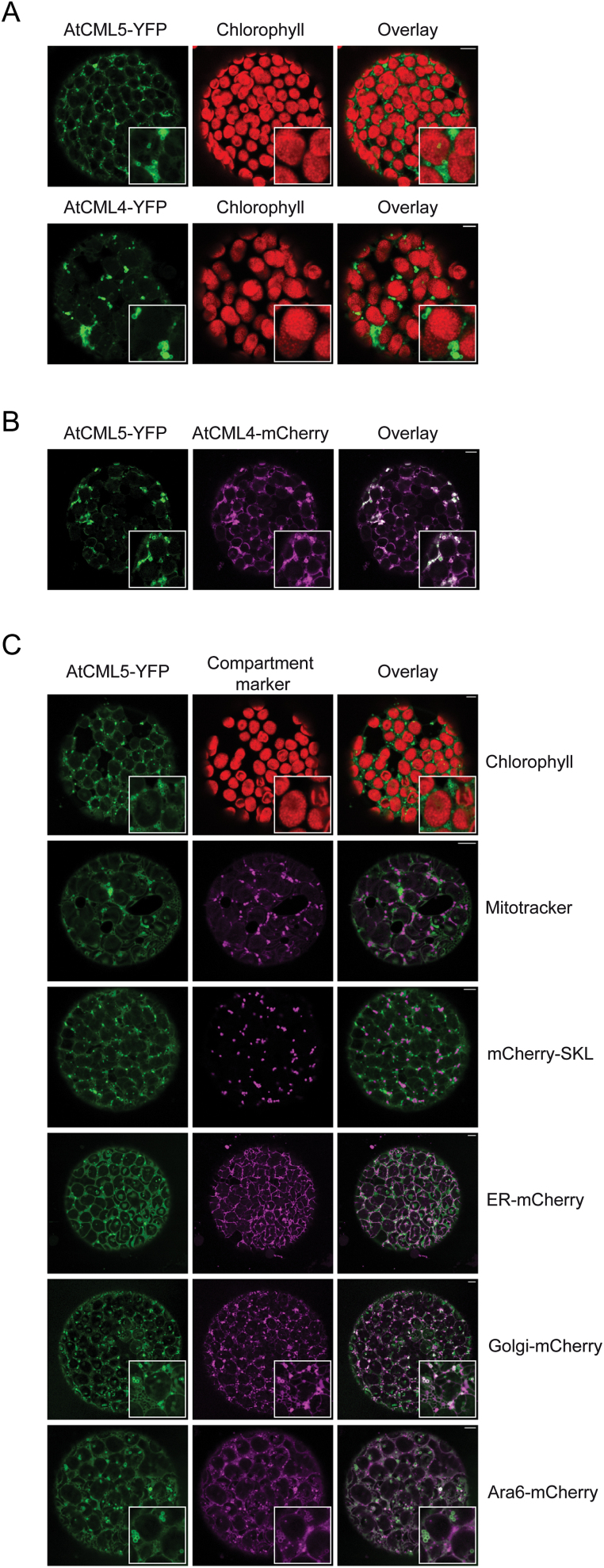
Fluorescence analysis of tobacco protoplasts transformed (**A**) individually with AtCML5-YFP and AtCML4-YFP and (**B**) co-transformed with AtCML5-YFP and AtCML4-mCherry. (**C**) Subcellular localization of AtCML5 elucidated by staining of mitochondria with Mito-Tracker Red CMXRos (mitotracker) or co-transformation of tobacco protoplasts with mCherry-SKL, ER-mCherry, Golgi-mCherry, and ARA6-mCherry. YFP fluorescence is depicted in green, Mito-Tracker and mCherry fluorescence in magenta, and chlorophyll fluorescence in red. The white bar within all images represents 5 µm. Enlargements are shown as inlays.

To get a more refined view on the subcellular location of AtCML4 and AtCML5, their subcellular localization was analysed *in planta* by their transient expression as C-terminal fusions to YFP in tobacco leaf cells. Laser scanning confocal microscopy of protoplasts prepared from transformed leaf cells 48h after *Agrobacterium* infiltration showed the fluorescence signal of both proteins in small spherical structures that appeared throughout the cytoplasm of the cell (Fig. 2A). Enlargement of these structures revealed a circular pattern (Fig. 2A, inlays) and co-transformation of AtCML5-YFP and AtCML4-mCherry showed that the two proteins co-localize (Fig. 2B). In light of this finding paired with the high sequence similarity of AtCML4 and AtCML5, the majority of further analyses was performed with AtCML5.

The AtCML5-YFP signal showed no overlap with chlorophyll fluorescence, demonstrating that AtCML5-YFP did not localize to the chloroplasts; it was also absent from the nucleus and the vacuole (Fig. 2C). Labelling of AtCML5-YFP–expressing protoplasts with Mito-Tracker Red CMXRos and co-expression analysis with an mCherry-tagged marker protein for peroxisomes (mCherry-SKL; Chigri *et al.*, 2012) revealed no overlap in fluorescence, also excluding the mitochondria and peroxisomes as the potential target compartments of AtCML5-YFP (Fig. 2C). Furthermore, co-expression analyses were performed with established marker proteins that label the endoplasmic reticulum (ER) (AtWAK2), Golgi (GmMAN1), and multivesicular bodies (MVBs)/endosomes (ARA6), respectively (Ueda *et al.*, 2001; Nelson *et al.*, 2007; Geldner *et al.*, 2009). ARA6 and GmMAN1 both displayed a predominantly dot-like signal as described previously and as seen exclusively in single transformations (Supplementary Fig. S1A). However, upon co-transformation with AtCML5-YFP, spherical structures became evident for both proteins. Moreover, a partial but not complete overlap with the signal from AtCML5-YFP was observed for both marker proteins exclusively in these spherical structures (Fig. 2C). Together, these results indicate that AtCML5 resides in vesicular structures within the interphase between the Golgi and the MVBs/endosomes, and circulation dynamics within these endomembrane compartments are the likely cause for the partial co-localization. Importantly, because transient expression of YFP-tagged AtCML4 and AtCML5 in Arabidopsis protoplasts controlled by their respective endogenous promoters results in a similar fluorescence signal pattern (Supplementary Fig. S1B), the observed signal in tobacco protoplasts is not an artefact caused by 35S-promoter driven overexpression.

**Fig. 3.**
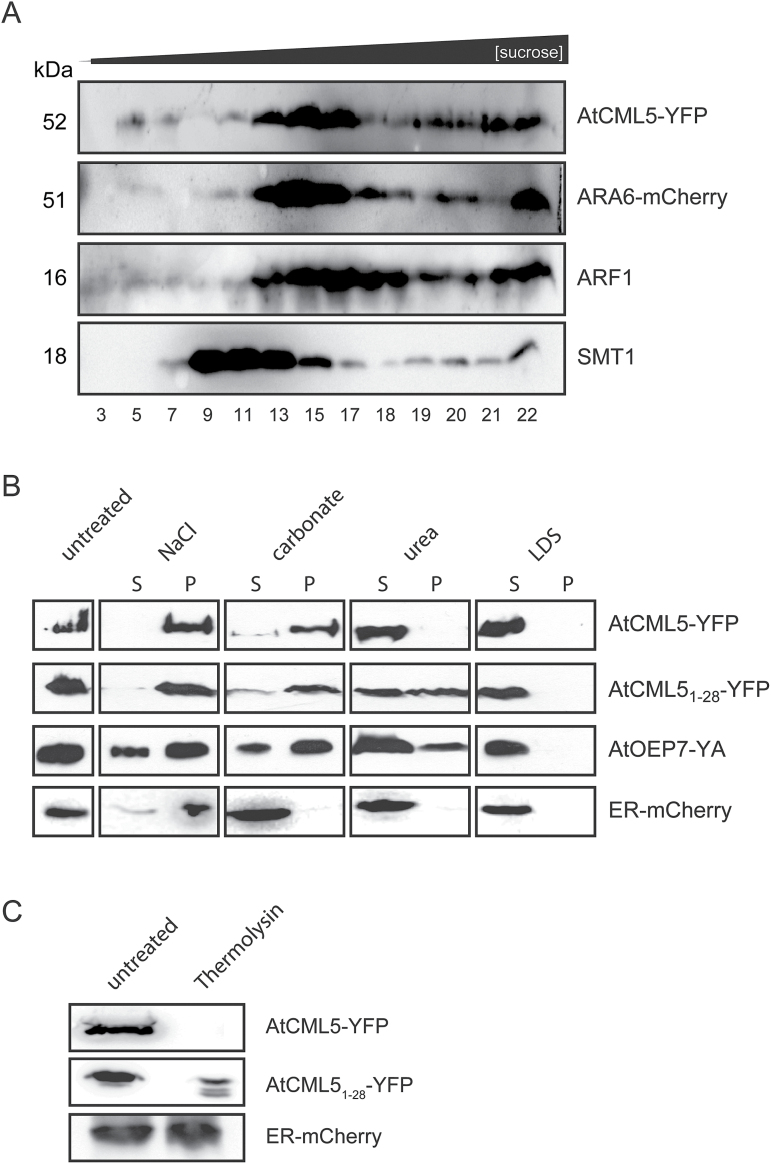
(**A**) Separation of isolated microsomes expressing AtCML5-YFP and ARA6-mCherry by sucrose density gradient centrifugation. Top-down aliquots of fractions as indicated were separated by SDS-PAGE and analysed by western blot using α-GFP to detect AtCML5-YFP, α-RFP to detect ARA6-mCherry, and antibodies against ARF1 and SMT1 to detect endogenous proteins of the Golgi and ER, respectively. (**B**) Extraction of membrane proteins using isolated microsomes from tobacco leaf cells expressing AtCML5-YFP, AtCML5_1–28_-YFP, and ER-mCherry or chloroplasts from tobacco leaf cells expressing AtOEP7-YA. Extraction was performed with 0.5M NaCl, 100mM Na_2_CO_3_ (pH 11.5; carbonate), 6M urea, or 2% LDS. Aliquots of supernatant and pellet fractions were separated by SDS-PAGE and proteins were visualized by western blot using tag-specific antibodies. (**C**) Thermolysin treatment of microsomes from tobacco leaf cells expressing AtCML5-YFP, AtCML5_1–28_-YFP, or ER-mCherry. Proteins were separated by SDS-PAGE and proteins were visualized by western blot using tag-specific antibodies.

To further confirm these findings, microsome fractions from tobacco leaf cells expressing either AtCML5-YFP or ARA6-mCherry were mixed after isolation and further separated on a sucrose density gradient. Using this approach, any influence of the expression of AtCML5 on the localization of ARA6 and vice versa was avoided. All fractions were separated by SDS-PAGE (Coomassie staining is shown in Supplementary Fig. S2A) and subsequently probed with antibodies against GFP and RFP to detect AtCML5-YFP and ARA6-mCherry, respectively (Fig. 3A). Antibodies against sterol methyltransferase 1 (SMT1), an ER integral membrane protein (Boutté *et al.*, 2010), and ADP-ribosylation factor 1 (ARF1), a protein found in the Golgi and the trans-Golgi network (Matheson *et al.*, 2008), were used to detect endogenous proteins of these compartments (Fig. 3A). While SMT1 was clearly separated from the other proteins, ARF1, ARA6, and AtCML5 signals were all found distributed in the same fractions of the gradient (Fig. 3A), supporting the dynamic localization of AtCML5.

### An N-terminal signal anchor sequence promotes targeting of AtCML5 to the endomembrane system and its stable association with endosomal membranes

**Fig. 4.**
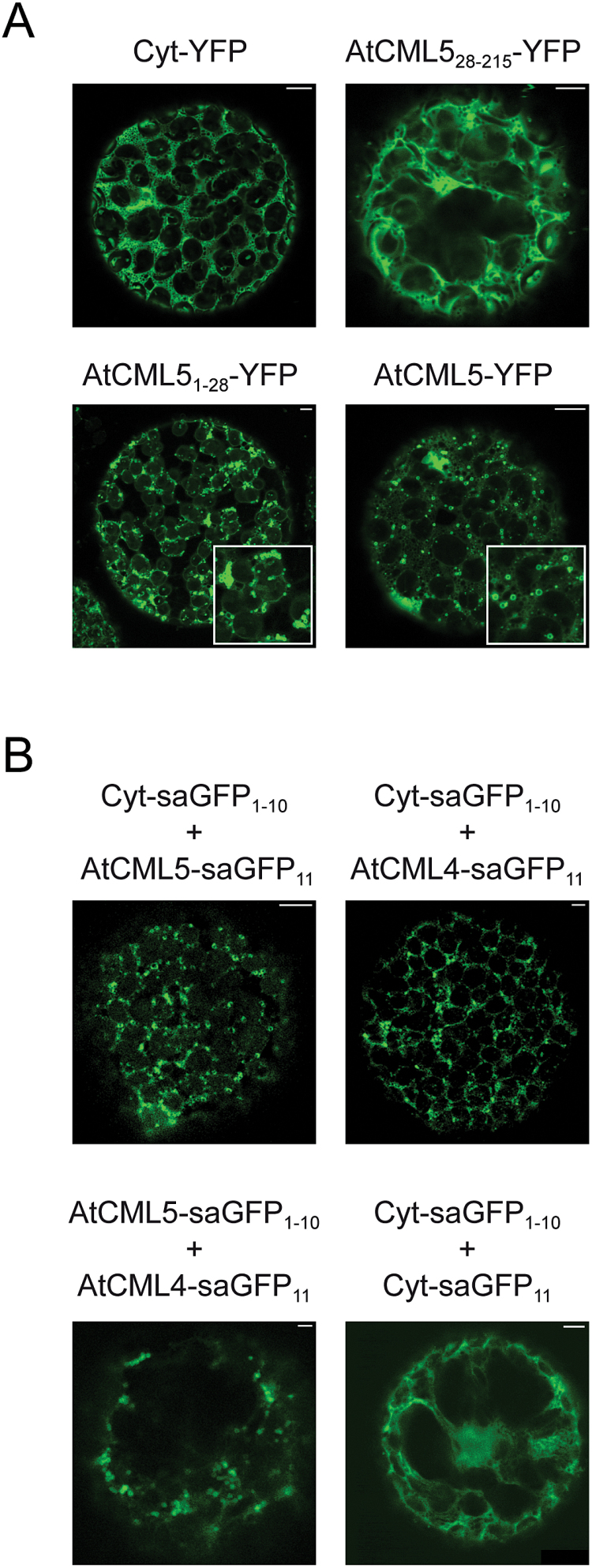
(**A**) Fluorescence analysis of tobacco leaf cell protoplasts transformed with AtCML5-YFP, AtCML5_1–28_-YFP, AtCML5_28–215_-YFP and a cytosolic marker (Cyt-YFP). (**B**) Fluorescence analysis of tobacco leaf cell protoplasts co-transformed with the saGFP pairs AtCML5-saGFP_11_ + Cyt-saGFP_1–10_, AtCML4-saGFP_11_ + Cyt-saGFP_1–10_, AtCML4-saGFP_11_ + AtCML5-saGFP_1–10_, and Cyt-saGFP_1–10_ + Cyt-saGFP_11_. The white bar within all images represents 5 µm.

In the next step, the question was addressed whether the *in silico* predicted N-terminal targeting signal indeed mediates subcellular targeting. A series of YFP-tagged truncated variants of AtCML5 was generated and their subcellular localization investigated (Fig. 4A). Notably, the variant lacking the first 28 amino acids (AtCML5_28–215_-YFP), which are strongly conserved between AtCML4 and AtCML5 (Fig. 1), showed a uniform fluorescence pattern devoid of any vesicular structures. The pattern is similar to that observed with cytosolic protein (Fig. 4A, Cyt-YFP), indicating that the protein is no longer targeted into the endomembrane system. By contrast, a variant comprising only the first 28 amino acids of AtCML5 (AtCML5_1–28_-YFP) showed the same spherical pattern as the full-length protein (Fig. 4A, AtCML5-YFP). These results strongly indicate that the first 28 amino acids of AtCML5 are both essential and sufficient for targeting of the protein into the endomembrane system.

The spherical pattern observed for the AtCML5-YFP fluorescence indicates that the protein is associated with the vesicle membrane rather than localized inside the vesicle lumen. In line with this observation, a set of programmes for transmembrane-domain prediction indicated a potential transmembrane helix in the N-terminus of both AtCML4 and AtCML5 that partially overlaps with the targeting sequence (Supplementary Table S5). This region therefore resembles the transmembrane domain of typical single-pass membrane proteins. To confirm the integration of AtCML5 into the membrane, extraction experiments using isolated microsomal fractions from tobacco cells expressing AtCML5-YFP or ER-mCherry (AtWAK2 is a luminal ER protein) were performed. As a control, chloroplast membranes isolated from tobacco cells expressing fluorescence-tagged chloroplast OEP7 from *A. thaliana* (AtOEP7-YA) were used, because OEP7 is also a single-pass membrane protein (Salomon *et al.*, 1990). Western blot analysis confirmed the presence of the tagged proteins in the microsomal or chloroplast membrane pellet (Fig. 3B, untreated). As expected, treatment of membranes with either 0.5 NaCl or alkaline carbonate (pH 11.5) could not extract the majority of the OEP7 protein from the membrane fraction. AtCML5 behaved in a manner very similar to OEP7, with only minor signals observed in the supernatant of the carbonate extraction (Fig. 3B, AtCML5-YFP). By contrast, AtWAK2 was found in the supernatant after alkaline carbonate treatment (Fig. 3B, ER-mCherry) because it converts vesicles into sheets and thus releases their soluble content as well as proteins peripherally associated with the membrane. Only integral proteins remain associated with the membrane under these conditions (Fujiki *et al.*, 1982). Control treatments with 6M urea or LDS resulted in a release of all proteins into the soluble fraction. The same extraction experiments were performed with isolated microsomes from tobacco cells expressing the AtCML5_1–28_-YFP variant and demonstrated a very similar extraction pattern as AtCML5-YFP (Fig. 3B, AtCML5_1–28_-YFP). Together, these results strongly support AtCML5 as a single-pass membrane protein with an N-terminal signal anchor within its first 28 amino acids.

### The CaM domain of AtCML5 is exposed on the cytosolic surface

The finding that AtCML5 is anchored to the membrane raises the question of whether the C-terminal CaM domain is luminal or exposed to the cytosolic surface. To address this question, isolated microsomes expressing AtCML5-YFP, AtCML5_1–28_-YFP or ER-mCherry were treated with thermolysin before western blot analysis (Fig. 3C). Immunodecoration clearly showed that the luminal ER marker protein AtWAK2 is fully protected from degradation by thermolysin. By contrast, both AtCML5-YFP and AtCML5_1–28_-YFP can be degraded by the protease, indicating that the C-terminally fused YFP protein that reacts with the antibody and thus the C-terminal CaM domain of AtCML5 is exposed to the outside of the microsomal vesicles.

To confirm the topology of AtCML5, the saGFP system was employed (Cabantous *et al.*, 2005; Machettira *et al.*, 2011). In this system the first 10 beta-sheets (saGFP_1–10_) and the 11th beta-sheet (saGFP_11_) of GFP are fused to different proteins. Owing to the high affinity of these moieties, they self-assemble to a functional GFP if they are present in the same compartment, resulting in a fluorescence signal. To show that this system can mark a specific compartment if one of the partners is integral to the membrane with the saGFP domain exposed to the surface and the other partner is a cytosolic protein, OEP7-saGFP_1–10_ was transformed together with Cyt-saGFP_11_ into tobacco mesophyll cells. Their co-expression resulted in ring-like fluorescence signals around the chloroplasts (Supplementary Fig. S1C). Co-expression of two cytosolic proteins resulted in the characteristic diffuse signal throughout the cell (Fig. 4B, Cyt-saGFP_1–10_ + Cyt-saGFP_11_). When AtCML5-saGFP_11_ was co-transformed with Cyt-saGFP_1–10_, a clear GFP signal could be observed in spherical structures like those observed for AtCML5-YFP (compare Fig. 4A, AtCML5-YFP with Fig. 4B, Cyt-saGFP_1–10_ + CML5-saGFP_11_). Co-expression of AtCML4 with the cytosolic marker (Fig. 4B, Cyt-saGFP_1–10_ + AtCML4-saGFP_11_) or AtCML5 (Fig. 4B, AtCML5-saGFP_1–10_ + AtCML4-saGFP_11_) also resulted in the typical spherical structures, confirming that AtCML4 and AtCML5 share the same localization and topology.

### AtCML4 and AtCML5 have typical properties of CaM-like Ca^2+^ sensors

Outside their N-terminal extension, AtCML4 and AtCML5 contain a CaM domain with high sequence similarity to canonical CaMs (cf. Fig. 1A), including four EF-hands with their characteristic 29-residue helix-loop-helix topology. The residues involved in Ca^2+^ coordination (D1, D/N3, D5, and E12) as well as the small G6 required for the conformational flexibility of the backbone are all conserved in these CMLs (http://pfam.xfam.org/family/PF00036, accessed 4 March 2016). When analysed *in vitro*, recombinant AtCML4 and AtCML5 lacking the signal-anchor sequence (AtCML4_27–194_-6His or AtCML5_27–215_-6His) displayed a faster migration on SDS-PAGE in the presence of Ca^2+^ compared to the same sample run in the absence of Ca^2+^ (Supplementary Fig. S3A). This characteristic, a Ca^2+^ -dependent mobility shift upon SDS-PAGE separation, has previously been associated with binding of calcium ions to the EF-hands of CaMs (Garrigos *et al.*, 1991; Maune *et al.*, 1992). AtCML5_27–215_-6His furthermore binds to phenyl-Sepharose in the presence of Ca^2+^ and can be eluted when Ca^2+^ is replaced by EDTA/EGTA (Supplementary Fig. S3B). These are both characteristic features of canonical CaMs and indicate that AtCML4 and AtCML5 can act as typical CaM proteins (Kursula, 2014).

### Phylogenetic distribution of CML4/5-like proteins

**Fig. 5.**
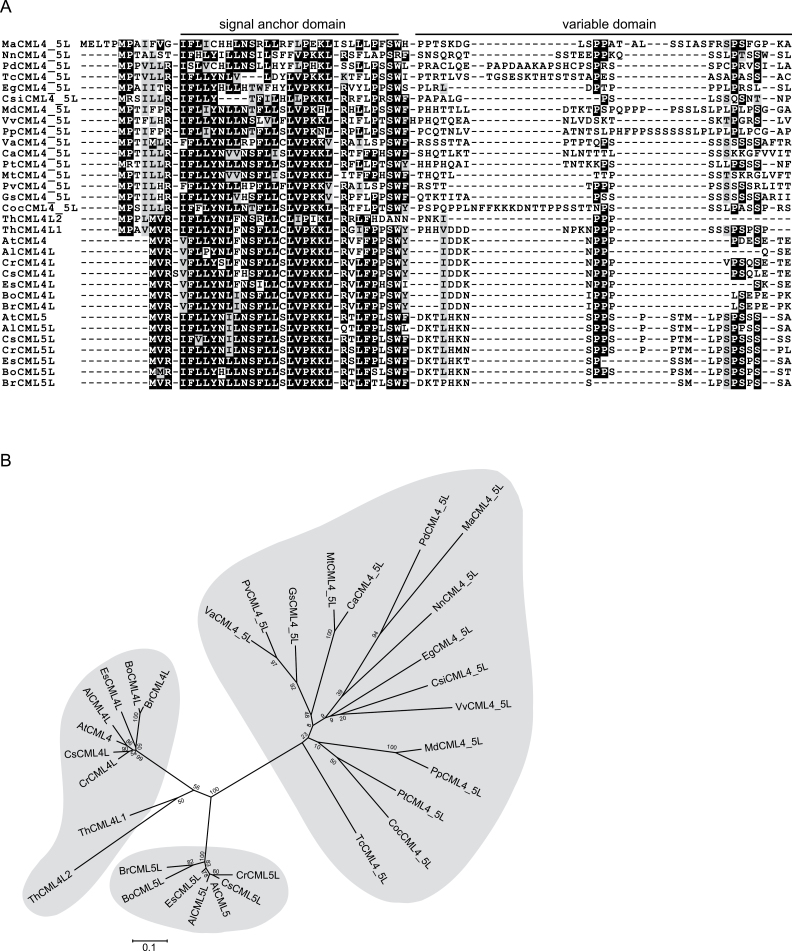
(**A**) Amino acid sequence alignment of the N-terminal extension of CML4/5 proteins from species representing different orders of flowering plants. Black boxes indicate identical amino acid residues while grey boxes indicate similar amino acids. (**B**) Phylogenetic relationship of CML4/5-like proteins from different orders of flowering plants. Phylogenetic tree construction was performed by maximum likelihood based on the sequence alignment provided in Supplementary Fig. S4. For accession numbers see Supplementary Table S3. A distribution of plants with CML4/5-like proteins within the different orders of angiosperms can be found in Supplementary Fig. S5.

Plants contain a vast number of different CMLs, indicating a strong diversification of this calcium sensor family (Zielinski, 1998; McCormack and Braam, 2003). They fall into various subfamilies that appear to be differentially distributed throughout the plant kingdom (Boonburapong and Buaboocha, 2007; Zhu *et al.*, 2015). Members of the CML2–7 clade (part of subfamily VII) are found exclusively in flowering plants (Zhu *et al.*, 2015). Unfortunately, the identification of orthologues for AtCML4 and AtCML5 among these proteins was substantially hindered by the generally very high sequence similarity of the CaM domain among all members of the CML2–7 clade. This is most likely a result of a functional constraint restricting the evolution of this domain, which renders it unsuitable for a high-resolution inference of evolutionary relationships among the homologues. Therefore, we used the presence of an N-terminal extension as the indicative characteristic to assign sequences as putative members of the CML4/5 subfamily. Using this restriction, CML4/5-like proteins could not be identified in the early branching magnoliophyte, *Amborella trichopoda*, and only two CML4/5-like proteins were found in the monocots. A CML4/5-like protein was found in the basal eudicot *Nelumbo nucifera* and at least one ortholog was found in core eudicots. The N-terminal extensions of all CML4/5-like proteins display sequence similarity to both AtCML4 and AtCML5 (Fig. 5A). In particular, the region of the signal anchor sequence is highly conserved, suggesting that all these proteins might be targeted into the endomembrane system. To trace the evolutionary history of this gene family, we performed a phylogenetic analysis on a representative subset of CML4/5 proteins spanning the full taxonomic diversity of plants where these proteins have been identified (Fig. 5B and Supplementary Fig. S4). The resulting tree places the sequences from the Brassicaceae in two well-supported monophyletic clades for CML4 and CML5, respectively (Fig. 5B). Moreover, all Brassicaceae CML4 and CML5 sequences share a common ancestry to the exclusion of the CML4/5 proteins from other plants. This indicates that the gene duplication event that gave rise to contemporary CML4 and CML5 most likely occurred in the last common ancestor of the Brassicaceae.

## Discussion

The emerging picture so far suggests that CMLs function in many different cellular processes, including various aspects of development as well as abiotic and biotic stress responses (reviewed in Bender and Snedden, 2013). Plants have allegedly extended their content of CaM-type sensors because their sessile lifestyle requires a higher degree of response to environmental changes. In addition to functional redundancy, the vast number of CMLs allows the detection of a larger variety of signals and a higher degree of tissue and developmental stage specificity, as well as subcellular targeting to include more cellular compartments in the Ca^2+^/CaM signalling network. Consequently, CMLs have been identified in compartments other than the cytoplasm, including two CMLs targeted into mitochondria and peroxisomes by means of N- or C-terminal sequence extensions (Chigri *et al.*, 2012; Bender and Snedden, 2013).

AtCML4 and AtCML5, two closely related CMLs from subfamily VII, possess an N-terminal sequence extension not found in canonical CaM that resembles a signal sequence for the secretion pathway (Fig. 1A and Supplementary Table S4). Upon transient expression in tobacco leaves under the 35S-promoters, AtCML4 and AtCML5 co-localized in spherical structures (Fig. 2A) and co-expression of AtCML5-YFP with marker proteins for Golgi (GmMan1) and MVBs/endosomes (ARA6) revealed a partial but not complete overlap (Fig. 2C). These results indicate that AtCML5 is localized within the endomembrane system, somewhere in the interface between Golgi and the endosomal system. Importantly, because transient expression of GFP-tagged AtCML4 and AtCML5 in Arabidopsis protoplasts controlled by their respective endogenous promoters results in a similar fluorescence signal pattern (Fig. S1B), the observed signal in tobacco protoplasts is not an artefact caused by 35S-promoter driven overexpression.

The partial overlap with ARA6 und GmMan1 occurred exclusively in spherical structures that are characteristic of AtCML5. Such large spherical structures are not typical for the endomembrane system and, in single transformations, ARA6-mCherry and Golgi-mCherry both displayed a solely punctuate fluorescence signal (Supplementary Fig. S1A). A similar phenotype was described for ARA7, a Rab5 homologue, expressed in a *gnom* mutant from *A. thaliana* (Ueda *et al.*, 2004). In the *gnom* mutant, Ara7-GFP tagged endosomes are no longer small dots but instead appear as clusters of ring-shaped structures. The authors attributed these abnormally deformed endosomes to the loss of GNOM. In the current study, a similar clustering of the ring-shaped structures was observed upon overexpression of AtCML5-YFP. This suggests that overexpression of AtCML5-YFP might affect the endosomal structure similarly to the loss of GNOM. GNOM encodes an endosomal GDP/GTP exchange factor for Arf GTPases that regulate vesicle formation (Geldner *et al.*, 2003) and a recent study has shown that it resides in distinct subdomains on Golgi cisternae and might be involved in maintaining trans-Golgi network/endosome function (Naramoto *et al.*, 2014). GNOM itself appears in ring-shaped structures upon expression as a GFP fusion (Naramoto *et al.*, 2014), similar to the spherical structures observed in the AtCML5-YFP expressing cells.

Proteins such as ARA6 associate with endosomal membranes by post-translational modifications, such as S-acylation, N-myristoylation, and prenylation (Running, 2014). Other proteins enter the secretory pathway co-translationally. They are initially translocated into the ER and then further routed through the Golgi. It seems likely that AtCML4 and AtCML5 reach their destination by this route. The initial 28 amino acids of their sequence is clearly recognized by many prediction programmes as a potential targeting sequence for the secretory pathway (Supplementary Table S4) and this part of the protein is sufficient for targeting of AtCML5 (Fig. 4A). Translocation through the ER is also supported by the weak background signal of AtCML5-YFP overlapping the ER-mCherry signal upon co-expression (Fig. 2C). However, in line with the lack of an ER-retention signal, AtCML4 and AtCML5 do not remain in the ER but are transferred further within the endomembrane system.

Neither AtCML4 nor AtCML5 are fully translocated into the lumen of the ER. Instead they are anchored in the membrane by an N-terminal signal anchor sequence. Both the protease protection assays and saGFP analysis suggest a topology with the C-terminal CaM-like domain exposed on the cytosolic surface (Figs 3C and 4B). This topology is strongly supported by the structure of their signal anchor sequence (Fig. 1B). Insertion orientation of single-pass membrane proteins integrates parameters such as charge difference across a transmembrane segment, its total hydrophobicity, and its hydrophobicity gradient (Engelman *et al.*, 1986; Harley *et al.*, 1998; von Heijne, 1994). A hydrophobicity gradient from the N- to the C-terminus of the transmembrane domain promotes insertion in an N_luminal_–C_cyt_ orientation, that is, the most hydrophobic terminus is preferentially translocated. Such a gradient exists in the deduced signal anchor sequence of both AtCML4 and AtCML5 (Fig. 1B). Furthermore, a single positively charged amino acid precedes the hydrophobic region of AtCML4 and AtCML5, while three positively charged amino acids are found directly after proline-20, which assumedly marks the end of the hydrophobic transmembrane helices (Fig. 1B).

In the absence of positive transport signals, such as ER retention signals or vacuolar sorting signals, localization of a protein within the endomembrane system may result from the properties of the transmembrane domain and its interaction with the membranes. Such a sorting mechanism is supported for AtCML5 by the fact that the first 28 amino acids of AtCML5 are both sufficient and necessary to translocate the protein to its destined compartment (Fig. 4A). However, the exact features that determine the final localization of AtCML4 and AtCML5 within the endomembrane system so far remain unknown.

Extensive searches could not identify orthologues to AtCML4 and AtCML5 in algae, mosses, or ferns nor in a representative of the basal Magnoliophyta, *Amborella trichopoda*. By contrast, CML4/5-like proteins seem to be universally present in the core eudicots as well as in the basal eudicot *Nelumbo nucifera*, and they are also found in the monocots *Musa acuminata* and *Phoenix dactylifera* (Fig. 5A, B and Supplementary Fig. S5). The presence of two paralogues of the CML4 and CML5 variety seems to be common to all Brassicaceae, but is not yet established in *Tarenaya hassleriana*, a Brassicales member of the Cleomaceae family. This suggests that CML4 and CML5 evolved from a gene duplication event that occurred early in the evolution of the Brassicaceae family. So far, it is unclear whether they are simply redundant or part of a further functional diversification of the CMLs within the Brassicaceae family. In Arabidopsis, AtCML5 is slightly higher expressed and shows a bit more variation in expression pattern than AtCML4; however, no distinctive function can be deduced from the expression data currently available in databases such as ‘Genevestigator’ (Hruz *et al.*, 2008). In all cases, the N-terminal extensions of the CML4/5-like proteins are highly conserved, especially within the potential signal anchor sequence, indicating that they are all localized in the endomembrane system (Fig. 5A). CML4/5-like proteins appear to be absent in some monocots such as maize or rice. However, the allocation of CML orthologues from other plants to the subfamilies and even more to individual members defined for Arabidopsis is often ambiguous, and CMLs with an N-terminal sequence extension exist in these plants. Future studies will have to elucidate whether (i) CML4/5-like proteins are truly absent in the genomes of these plants; (ii) these plants can substitute for the lack of CML4/5 by other means, such as the addition of a signal anchor sequence to a CML from a different class; or (iii) the specific function of CML4/5 is indeed not required in all flowering plants.

So what could be the function of a CML that is localized in the endomembrane system, somewhere in the interphase between the Golgi and the MVBs/endosomes? While the plant endomembrane system has strong similarities to those of animals and yeast, it also has some unique features, especially with regard to the heterogeneous endosomal compartments (reviewed in Contento and Bassham, 2012). One reason is the additional role that plant endosomes play in the maintenance of the vacuole and in cell growth, including the formation of the cell wall. In the latter function they are an important part of plant cell division, which occurs via the recruitment of cell wall material to the division plane. The CaM domain of AtCML4 and AtCML5 is exposed to the cytoplasm (Fig. 4B), suggesting that it recognizes cytosolic Ca^2+^ signals. Calcium has been well investigated as a component of intra-cellular membrane fusion reactions (reviewed in Hay, 2007) and it has been shown that homotypic membrane fusion in animals is affected by CaM antagonists (Pryor *et al.*, 2000). The vesicle binding factor early endosomal antigen 1 (EEA1) possesses a CaM binding motif (IQ domain), and SNARE proteins such as VAML2 and syntaxin13 have been shown to bind CaM (Mills *et al.*, 2001; De Haro *et al.*, 2003). It was thus suggested for the animal system that CaM is important for homotypic membrane fusion within the endomembrane system and is recruited to the membrane by EEA1 and syntaxin13 (Mills *et al.*, 2001). Plants do not contain any homologues of EEA1, therefore it is possible that membrane-anchored CMLs substitute directly for EEA1-mediated recruitment of CaM to the membrane. A role in vesicle fusion would also be in accordance with the impression that the spherical structures observed upon AtCML5-YFP expression are similar to those marked by GNOM (Naramoto *et al.*, 2014) and observed for ARA7 in the *gnom* mutant (Ueda *et al.*, 2004). A similar dilation of endosomal vesicles was also observed when wild-type plants were treated with Wortmannin, an inhibitor of phosphatidylinositol-3 kinase, a protein involved in vesicle formation (Robinson *et al.*, 2008). AtCML4 and AtCML5 could thus provide a potential tie to the function of the endomembrane system in hormone distribution and defence signalling pathways via the endomembrane system, where the involvement of Ca^2+^ signalling is well described. However, *in vivo* studies, for example, by use of loss-of-function mutants, will be required to elucidate the precise role of CML4/5-like proteins in plants.

## Supplementary data

Supplementary data are available at *JXB* online.

Fig. S1. Fluorescence analyses of (**A**) Tobacco protoplasts prepared from leaf mesophyll cells expressing ARA6-mCherry and Golgi-mCherry; (**B**) Arabidopsis protoplasts transformed with AtCML5-YFP and AtCML4-YFP expressed under their respective endogenous promoters, and (**C**) tobacco protoplasts prepared from leaf mesophyll cells co-expressing OEP7-saGFP_11_ + Cyt-saGFP_1–10_.

Fig. S2. (**A**) Coomassie staining of SDS-PAGEs showing sucrose gradient fractions and (**B**) full-size western blot for the thermolysin treatment in Fig. 3C.

Fig. S3. Ca^2+^-dependent mobility shift and phenyl-Sepharose binding assays.

Fig. S4. Box-shaded sequence alignment of CML4/5-like proteins from flowering plants.

Fig. S5. Distribution of CML4/5-like proteins within the phylogenetic tree of flowering plants.

Table S1. List of primer sequences.

Table S2. List of constructs used in this work.

Table S3. List of accession numbers used in sequence alignments and phylogenetic tree analysis.

Table S4. Targeting prediction analysis of AtCML4 and AtCML5.

Table S5. Transmembrane-domain prediction for AtCML4 and AtCML5.

Supplementary Data

## Abbreviations:

CaM: calmodulin
CML: calmodulin-like protein
ER: endoplasmic reticulum
MVBs: multivesicular bodies
saGFP: self-assembly GFP.
