# Supplementary material for: The calmodulin-like proteins AtCML4 and AtCML5 are single-pass membrane proteins targeted to the endomembrane system by an N-terminal signal anchor sequence
Source: J Exp Bot. 2016 Mar 29;67(13):3985–96. doi: 10.1093/jxb/erw101 (PMC4915527; doi:10.1093/jxb/erw101)
Supplement: Supplementary Data [file supp_67_13_3985__index.html]

The calmodulin-like proteins AtCML4 and AtCML5 are single-pass membrane proteins targeted to the endomembrane system by an N-terminal signal anchor sequence — The calmodulin-like proteins AtCML4 and AtCML5 are single-pass membrane proteins targeted to the endomembrane system by an N-terminal signal anchor sequence — Supplementary Data 

# The calmodulin-like proteins AtCML4 and AtCML5 are single-pass membrane proteins targeted to the endomembrane system by an N-terminal signal anchor sequence

## Supplementary Data

Data files

- supplementary\_figures\_S1\_S5\_tables\_S1\_S5.pdf - Supplementary Data
