# Supplementary material for: The calmodulin-like proteins AtCML4 and AtCML5 are single-pass membrane proteins targeted to the endomembrane system by an N-terminal signal anchor sequence
Source: J Exp Bot. 2016 Mar 29;67(13):3985–96. doi: 10.1093/jxb/erw101 (PMC4915527; doi:10.1093/jxb/erw101)
Supplement: Supplementary Data [file supp_erw101_supplementary_figures_S1_S5_tables_S1_S5.pdf]

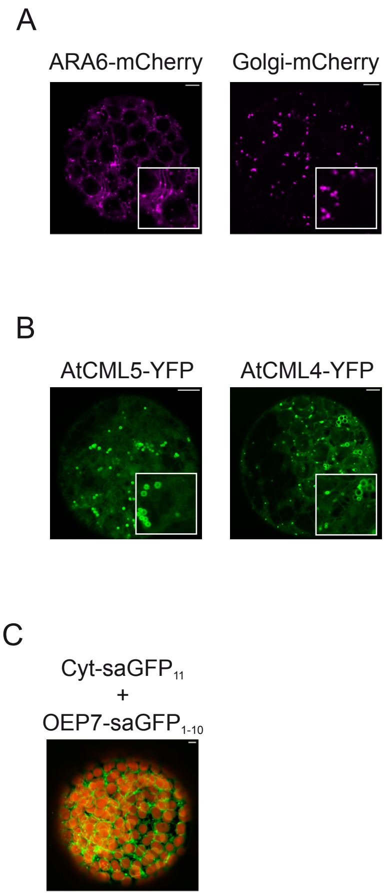

Figure S1. Fluorescence analysis of (A) Tobacco protoplasts prepared from leaf mesophyll cells expressing ARA6-mCherry and Golgi-mCherry; (B) Arabidopsis protoplasts transformed with AtCML5-YFP and AtCML4-YFP expressed under their respective endogenous promoters or (C) tobacco protoplasts prepared from leaf mesophyll cells co-expressing OEP7-saGFP$_{11}$+Cyt-saGFP$_{1\text{-}10}$.

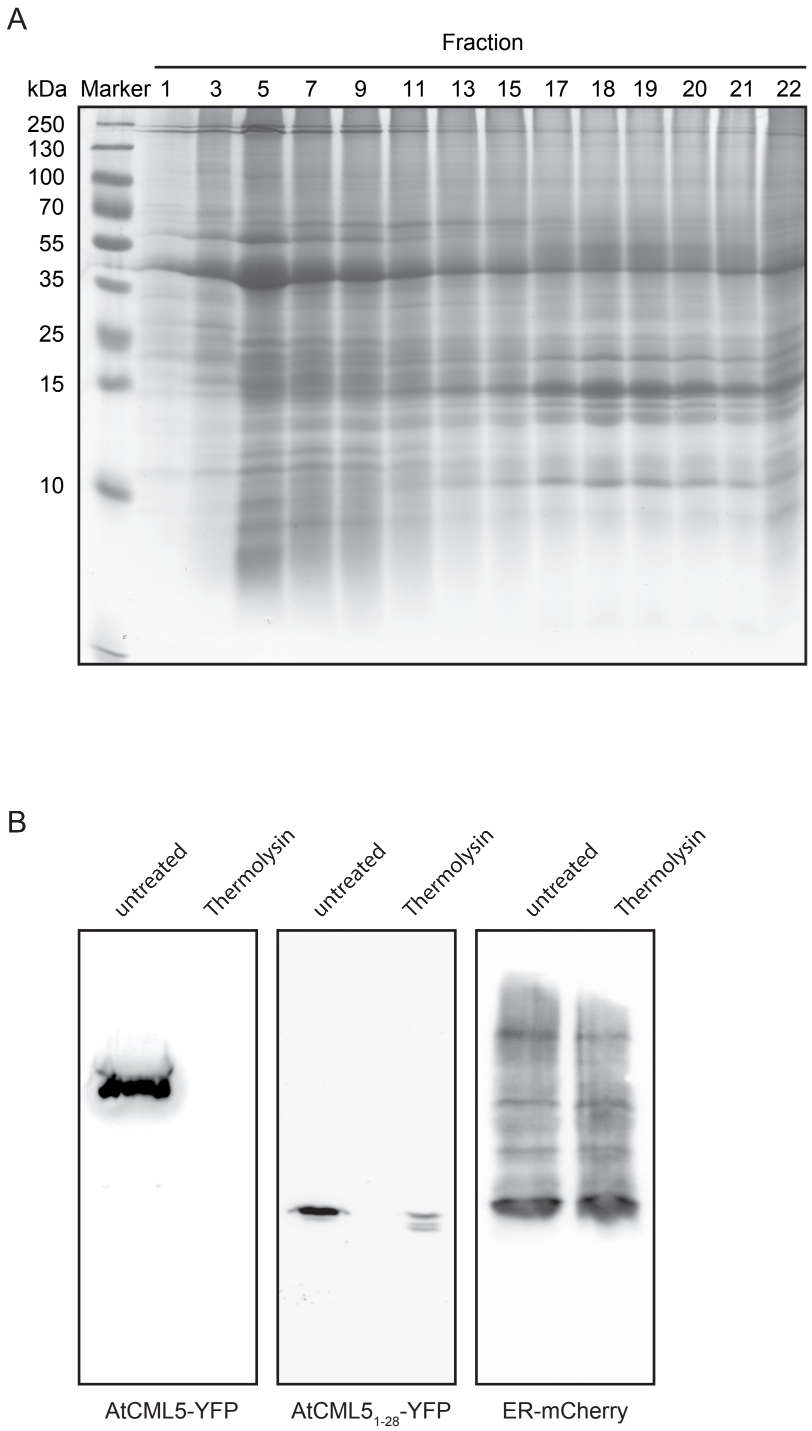

Figure S2. (A) SDS-PAGE separation and Coomassie staining of fractions from the sucrose density gradient of isolated microsomes. (B) Full-size western blot of the thermolysin treatment shown in Fig. 3C.

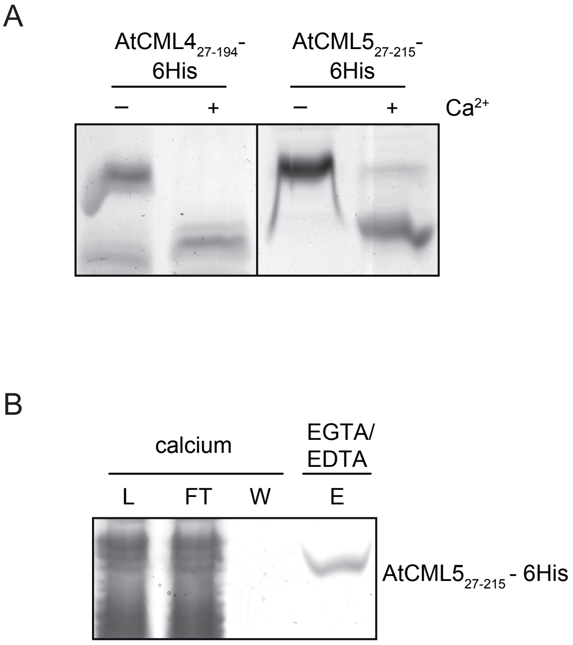

Figure S3. (A) Purified recombinant AtCML4$_{27-194}$-6His and AtCML5$_{27-215}$-6His were separated by SDS-PAGE in the presence or absence of calcium. Calcium-dependent shifts in electrophoretic mobility were analysed by SDS-PAGE and Coomassie staining. (B) Soluble extracts from *E. coli* cells expressing AtCML5$_{27-215}$-6His were loaded on phenyl-sepharose in the presence of calcium. Elution was performed by substituting EDTA for calcium and fractions were analysed by SDS-PAGE and Coomassie staining. L: load; FT: flow-through; W: wash; E: elution.

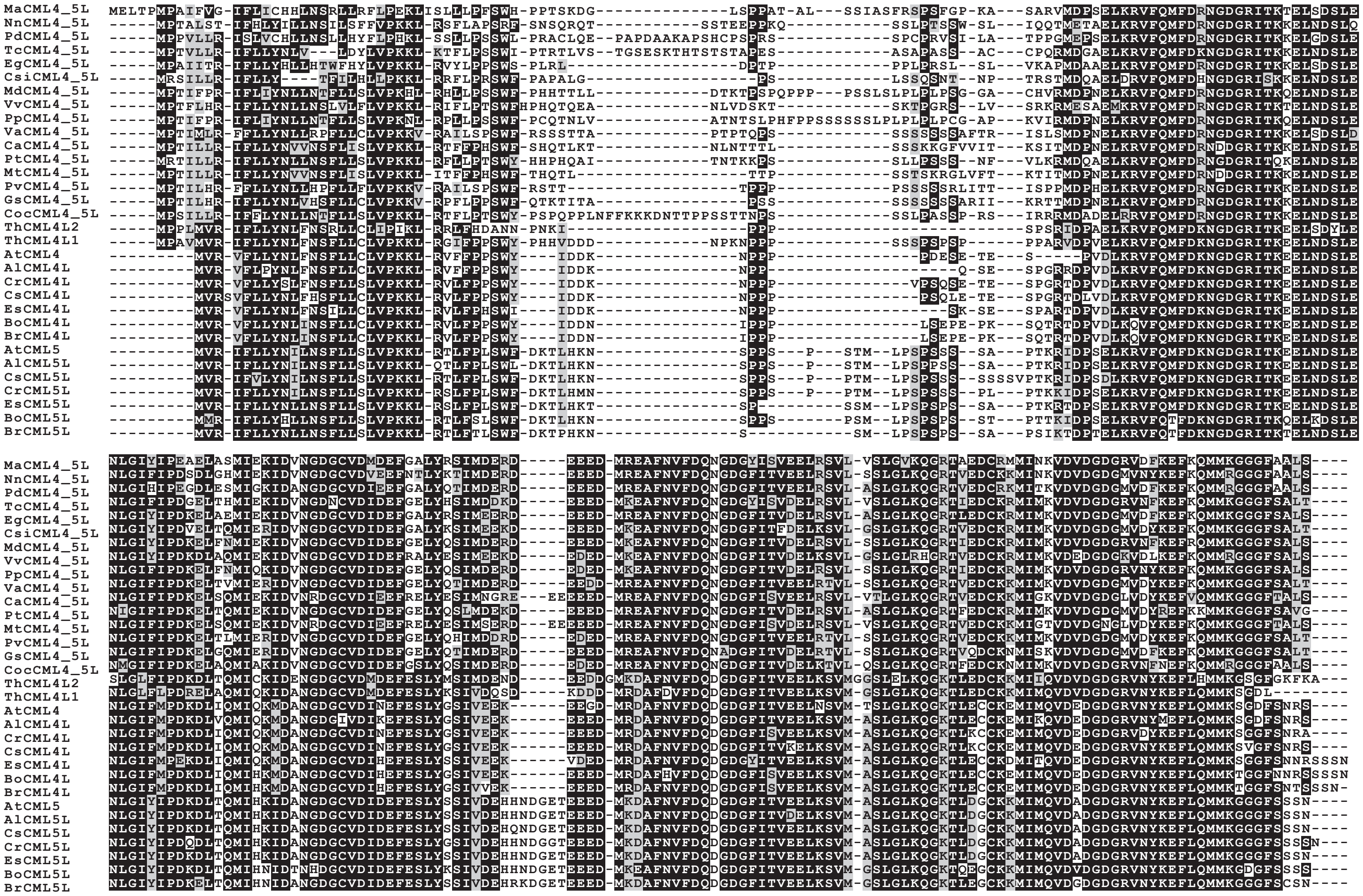

Figure S4. Amino acid sequence alignment of CML4_5 proteins from species representing different orders of flowering plants. Black boxes indicate identical amino acid residues while grey boxes indicate a conserved amino acid substitution.

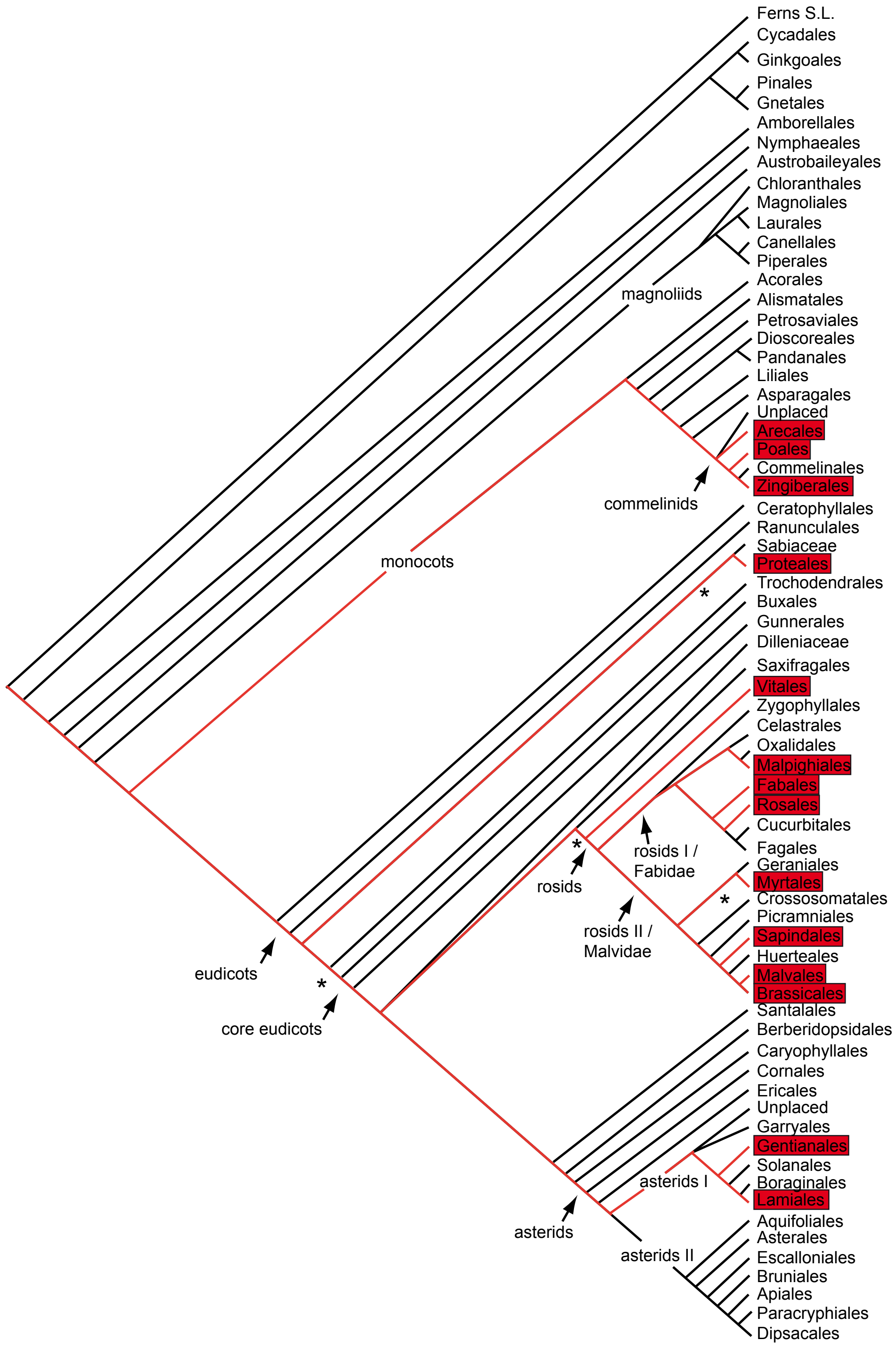

Figure S5. Distribution CML4_5-like proteins within the different orders of flowering plants. Orders in which CML4_5-like proteins were identified are marked by red branches and red boxes. Due to lack of full genome sequences covering all orders of plants, the absence of a marking does not indicate absence of CML4_5-like protein. The tree was constructed by the Angiosperm Phylogeny Website and used with the kind permission of the author (http://www.mobot.org/MOBOT/research/APweb/welcome.html).

Table S1. List of primer sequences

**Primer**	**Sequence (5‘->3‘)**
AtCML5$_{1-28}$NotI rv	AATGCGGCCGCCAAGAGGGAAAAGAGTTCG
AtCML5$_{27-215}$ApaI fw	ATGGGCCCATGCCTCTTTCTTGGTTCGAC
AtCML5 ApaI fw	CAGGGCCCATGGTGAGAATATTCCTTCTC
AtCML5 NotI rv	TTAGCGGCCGCCATTACTGCTGCTAAAG
AtCML5$_{27-215}$Nde1 fw	CATCATATGCCTCTTTCTTGGTTCGACAAAAC
AtCML5 Xho1 rv	TGTGCTCGAGATTACTGCTGCTAAAGCCACC
AtCML4$_{27-194}$Nde1 fw	CATCATATGCCTCCTTCTTGGTACATCGACG
AtCML4 Xho1 rv	TGTGCTCGAGTGATCTATTGCTAAAGTCACCAC
ARA6ApaI fw	TGGGCCCATGGGATGTGCTTCTTCTCTTC
ARA6NotI rv	AATGCGGCCGCCTGACGAAGGAGCAGGACG
AtCML4-BINfw	GAGGGCCCATGGTGAGAGTCTTTC
AtCML4-BINrv	TTAGCGGCCGCCTGATCTATTGCTAAAG
mCher_ApaI_fw	TCCGGGCCCATGGTGAGCAAGGGCG
mCherSKLnotIrv2	CGTTAGCGGCCGCTTACAATTTTGACTTGTACAGCTCGTC
1g66410 pBINfw	CCGGGCCCATGGCGGATCAG
1g66410 pBINrv	TTAGCGGCCGCCCTTAGCCATCATAATC
Fw_C4_PrC5_EcoRI	CTTGAATTCTTTTCTGTCTGAATCTCTG
Rv_C4_PrC5_XhoI	AAGCTCGAGAACTCTTGGCTTTG
Fw_CML4_XhoI	AAGCTCGAGATGGTGAGAGTCTTTC
Rv_CML4_NcoI	TCGCCCTTGCTCACCATGGCTGATCTATTGCTAAAGTC
Fw_YFP_C4_NcoI	GACTTTAGCAATAGATCAGCCATGGTGAGCAAGGGCGA
Rv_YFP_SpeI	AATCCTCGGACTAGTCTAGCGCCCGCTCTTGTAC
FwC5specPrC5EcoRI	GCAACTTGAATTCACATTTTTTCAGTTATTTTGTG
Rv_C5_PrC5_XhoI	AAGCTCGAGAACTGTTGAATCACAACTC
Fw_CML5_XhoI	GCCAAGCTCGAGATGGTGAGAATATTCCTTCTC
Rv_CML5_NcoI	TCGCCCTTGCTCACCATGGCATTACTGCTGCTAAAG
Fw_YFP_C5_NcoI	CTTTAGCAGCAGTAATGCCATGGTGAGCAAGGGCGA
Rv_YFP_SpeI	AATCCTCGGACTAGTCTAGCGCCCGCTCTTGTAC

Table S2. List of constructs used in this work

Construct	Vector	Reference
AtCML5-YFP	pBIN19	this publication
AtCML5$_{1-28}$-YFP	pBIN19	this publication
AtCML5$_{27-215}$-YFP	pBIN19	this publication
AtCML5$_{27-215}$-His	pET21b	this publication
AtCML4$_{27-194}$-His	pET21b	this publication
Ara6-mCherry	pBIN19	this publication
Golgi-mCherry	pBIN	Nelson et al. 2007
ER-mCherry	pBIN	Nelson et al. 2007
mCherry-SKL	pBIN	this publication
Cyt-YFP	pBIN19	this publication
OEP7-YFP-AEQ	pBIN19	Mehlmer et al. 2012
OEP7-saGFP$_{1-10C}$	pBIN19	this publication
Cyt- saGFP$_{1-10C}$	pBIN19	this publication
Cyt- saGFP$_{11C}$	pBIN19	this publication
AtCML5-saGFP$_{1-10C}$	pBIN19	this publication
AtCML5-saGFP$_{11C}$	pBIN19	this publication
AtCML4-saGFP$_{1-10C}$	pBIN19	this publication
AtCML4-saGFP$_{11C}$	pBIN19	this publication
cml5::AtCML5-YFP	pGREENII	this publication
cml4::AtCML4-YFP	pGREENII	this publication

Table S3. List of accession numbers and the corresponding species for sequences used in alignments and phylogenetic tree analysis

**Name**	**Accession number**	**Species**	**Order**
AlCML4L	XP_002876503.1	*Arabidopsis lyrata subsp. lyrata*	Brassicales
AlCML5L	XP_002881895.1	*Arabidopsis lyrata subsp. lyrata*	Brassicales
AtCaM4	NP_001185330.1	*Arabidopsis thaliana*	Brassicales
AtCML2	NP_193022.1	*Arabidopsis thaliana*	Brassicales
AtCML3	NP_187405.1	*Arabidopsis thaliana*	Brassicales
AtCML4	NP_191503.1	*Arabidopsis thaliana*	Brassicales
AtCML5	NP_565996.1	*Arabidopsis thaliana*	Brassicales
AtCML6	NP_192238.1	*Arabidopsis thaliana*	Brassicales
AtCML7	NP_172089.1	*Arabidopsis thaliana*	Brassicales
CrCML5L	XP_006296176.1	*Capsella rubella*	Brassicales
CsCML4L	XP_010469361.1	*Camelina sativa*	Brassicales
CsCML5L	XP_010508448.1	*Camelina sativa*	Brassicales
CsiCML4_5L	KDO46255.1	*Citrus sinensis*	Sapindales
EgCML4_5L	XP_010069294.1	*Eucalyptus grandis*	Myrtales
EsCML4L	XP_006402687.1	*Eutrema salsugineum*	Brassicales
EsCML5L	XP_006397493.1	*Eutrema salsugineum*	Brassicales
GsCML4_5L	KHN33309.1	*Glycine soja*	Fabales
MaCML4_5L	XP_009417008.1	*Musa acuminata subsp. malaccensis*	Zingiberales
MdCML4_5L	XP_008369144.1	*Malus domestica*	Rosales
MtCML4_5L	XP_003597517.1	*Medicago truncatula*	Fabales
NnCML4_5L	XP_010268140.1	*Nelumbo pentapetala*	Proteales
PdCML4_5L	XP_008791779.1	*Phoenix dactylifera*	Arecales
PpCML4_5L	XP_007227575.1	*Prunus persica*	Rosales
PtCML4_5L	XP_002310432.2	*Populus trichocarpa*	Malphigiales
PvCML4_5L	XP_007150430.1	*Phaseolus vulgaris*	Fabales
TcCML4_5L	XP_007033950.1	*Theobroma cacao*	Malvales
ThCML4L1	XP_010526084.1	*Tarenaya hassleriana*	Brassicales
ThCML4L2	XP_010525867.1	*Tarenaya hassleriana*	Brassicales
VaCML4_5L	KOM44447.1	*Vigna angularis*	Fabales
VvCML4_5L	XP_002266359.1	*Vitis vinifera*	Vitales

Table S4. Targeting prediction analysis for AtCML5 and AtCML4

The full length coding sequences of AtCML5 and AtCML4 were analysed on the web interphase of a number of different targeting prediction programs. The results of those programs predicting subcellular targeting for at least one of the two proteins are shown.

Prediction tool	AtCML5 (At2g43290)	AtCML4 (At3g59440)
**TargetP 1.1 (a)**	Secretory protein	Secretory protein
**Psort (b)**	ER membrane	Plasma membrane
**BaCelLo (c)**	Secretory protein	Secretory protein
**Predotar (d)**	ER	ER
**Yloc (e)**	Vacuole (38.9%), Cytoplasm (22.8%), Mitochondrion (17.3%)	Extracellular space (79.45 %)
**Golgi Predictor (f)**	Golgi (score 26.13; threshold 20)	Golgi (score 29.56; threshold 20)
**MemPype (g)**	Signal peptide (1-21aa) 93 % internal membrane score; no TMD	Signal peptide (1-19aa) 66% Internal membrane score; no TMD

(a) http://www.cbs.dtu.dk/services/TargetP/

(b) http://psort.hgc.jp/form.html

(c) http://gpcr.biocomp.unibo.it/bacello/

(d) https://urgi.versailles.inra.fr/predotar/predotar.html

(e) http://abi.inf.uni-tuebingen.de/Services/YLoc/webloc.cgi

(f) http://ccb.imb.uq.edu.au/golgi/golgi_predictor.shtml

(g) http://mu2py.biocomp.unibo.it/mempype/default/predict

Table S5. TM-domain predictions for AtCML5 and AtCML4

The full length coding sequences of AtCML5 and AtCML4 were analysed on the web interphase of different programs for TM-domain analysis.

Prediction tool	AtCML5 (At2g43290)	AtCML4 (At3g59440)
**DAS-TMfilter (a)**	Potential signal peptide, no TMD	Potential signal peptide, no TMD
**TMpred (b)**	TMD (1-19 aa)<br>N-terminus lumen (strong preference)	TMD (1-19 aa)<br>N-terminus lumen (strong preference)
**TopPred 1.10 (c)**	TMD predicted<br>(1-21 aa score 0.854,<br>39-59 aa score 0.617)	TMD predicted<br>(1-21 aa, score 0.944)

(a) http://www.enzim.hu/DAS/DAS.html

(b) http://www.ch.embnet.org/software/TMPRED_form.html

(c) http://mobyle.pasteur.fr/cgi-bin/portal.py#forms::toppred
